# Sequence-based identification of amyloidogenic β-hairpins reveals a prostatic acid phosphatase fragment promoting semen amyloid formation

**DOI:** 10.1016/j.csbj.2023.12.023

**Published:** 2023-12-21

**Authors:** Laetitia F. Heid, Emil Dandanell Agerschou, Asuka A. Orr, Tatsiana Kupreichyk, Walfried Schneider, Michael M. Wördehoff, Melanie Schwarten, Dieter Willbold, Phanourios Tamamis, Matthias Stoldt, Wolfgang Hoyer

**Affiliations:** a Institut für Physikalische Biologie, Heinrich Heine University Düsseldorf, 40204 Düsseldorf, Germany; b Artie McFerrin Department of Chemical Engineering, Texas A&M University, College Station, TX 77843-3122, United States; c Institute of Biological Information Processing (IBI-7) and JuStruct: Jülich Center for Structural Biology, Forschungszentrum Jülich, 52425 Jülich, Germany; d Department of Materials Science and Engineering, Texas A&M University, College Station, TX 77843-3033, United States

**Keywords:** Amyloid, β-hairpin, Protein misfolding, Protein aggregation, Proteopathy, Semen amyloid

## Abstract

β-Structure-rich amyloid fibrils are hallmarks of several diseases, including Alzheimer’s (AD), Parkinson’s (PD), and type 2 diabetes (T2D). While amyloid fibrils typically consist of parallel β-sheets, the anti-parallel β-hairpin is a structural motif accessible to amyloidogenic proteins in their monomeric and oligomeric states. Here, to investigate implications of β-hairpins in amyloid formation, potential β-hairpin-forming amyloidogenic segments in the human proteome were predicted based on sequence similarity with β-hairpins previously observed in Aβ, α-synuclein, and islet amyloid polypeptide, amyloidogenic proteins associated with AD, PD, and T2D, respectively. These three β-hairpins, established upon binding to the engineered binding protein β-wrapin AS10, are characterized by proximity of two sequence segments rich in hydrophobic and aromatic amino acids, with high β-aggregation scores according to the TANGO algorithm. Using these criteria, 2505 potential β-hairpin-forming amyloidogenic segments in 2098 human proteins were identified. Characterization of a test set of eight protein segments showed that seven assembled into Thioflavin T-positive aggregates and four formed β-hairpins in complex with AS10 according to NMR. One of those is a segment of prostatic acid phosphatase (PAP) comprising amino acids 185–208. PAP is naturally cleaved into fragments, including PAP(248−286) which forms functional amyloid in semen. We find that PAP(185−208) strongly decreases the protein concentrations required for fibril formation of PAP(248−286) and of another semen amyloid peptide, SEM1(86−107), indicating that it promotes nucleation of semen amyloids. In conclusion, β-hairpin-forming amyloidogenic protein segments could be identified in the human proteome with potential roles in functional or disease-related amyloid formation.

## Graphical Abstract

**Figure:**
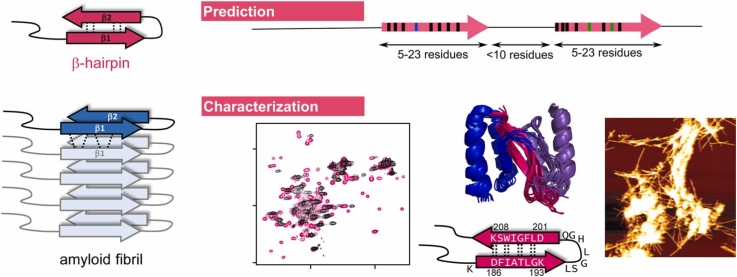

## 1 Introduction

**Fig. 1:**
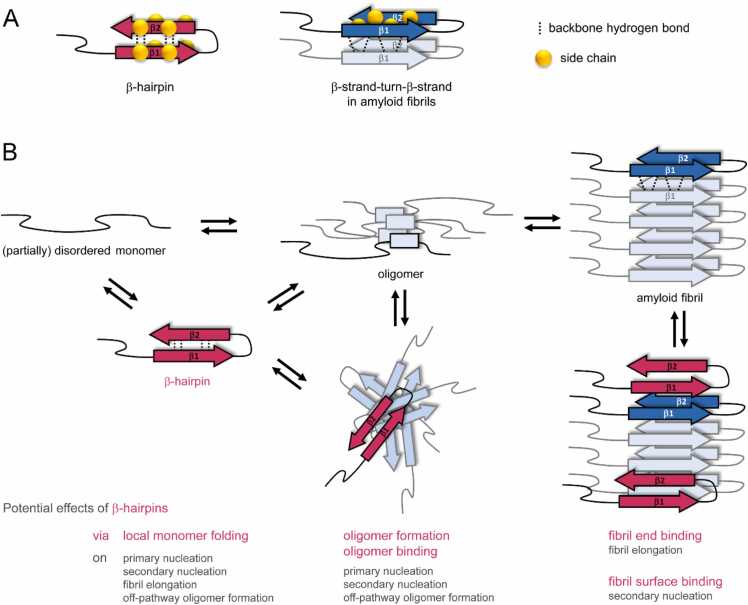
: Schematic of the potential involvement of β-hairpin conformers (red) in the amyloid formation reaction. (A) Comparison of β-hairpin with β-strand-turn-β-strand motif in amyloid fibrils. Backbone hydrogen bonds are intramolecular in β-hairpins but intermolecular in typical amyloid fibrils, resulting in antiparallel (β-hairpin) vs. parallel (amyloid fibril) β-sheets. For the β-strand-turn-β-strand motif selected side chains are displayed as spheres to illustrate intramolecular side chain-side chain interactions that stabilize amyloid fibrils in a steric zipper-like fashion. (B) β-Hairpins may have multiple effects in amyloid formation pathways.

Amyloid fibrils are a structural key feature of several prevalent diseases including Alzheimer’s disease (AD), Parkinson’s disease (PD), and type 2 diabetes (T2D), but also fulfill functional roles in biology [1], [2], [3], [4]. Their principal framework is the cross-β structure, in which β-strands oriented perpendicular to the fibril axis form long β-sheets, stabilized by backbone hydrogen bonds (Fig. 1A). Amyloid fibrils consist of two or more such β-sheets that pack against each other, supported by interactions between their side chains. In the fibrils of disease-related amyloidogenic proteins a parallel in-register β-sheet organization is commonly established, i.e., identical side chains of neighboring monomer units stack on top of each other [1], [4]. Fibrils grow by stepwise addition of monomers, which form multiple β-strand-kink-β-strand motifs. The different β-strand forming segments of one monomer can make contact with each other via side chain-side chain interactions (Fig. 1A). This β-strand-kink-β-strand conformation is not stable in free monomers, but is stabilized in the amyloid fibril by the hydrogen bond network and side chain interactions within the highly ordered intermolecular β-sheets.

A related structural motif is the β-hairpin (Fig. 1A, pink red) [5], [6]. In contrast to the β-strand-kink-β-strand conformation in amyloid fibrils, the β-hairpin features intramolecular backbone hydrogen-bonding within an antiparallel β-sheet. The β-hairpin conformation is readily accessible in monomeric peptides provided there is a sequence segment with turn-forming propensity connecting two stretches of residues with β-sheet propensity. The kinetic stability of a β-hairpin is largely determined by the side chain interactions among the β-sheet residues. Even in optimized β-hairpin peptides, the hairpin conformation is not persistent but folds and unfolds on the microsecond timescale [7]. Nevertheless, molecular dynamics (MD) simulations have identified a substantial fraction of β-hairpin-containing structures in the conformational ensembles of monomeric amyloidogenic intrinsically disordered proteins (IDPs), confirmed by experimental evidence [8], [9], [10], [11], [12], [13], [14], [15], [16], [17], [18], [19], [20], [21], [22], [23]. This observation can be explained by the presence of sequence segments with high β-sheet propensity, a determinant of high amyloidogenicity. Due to its preferred occurrence in amyloidogenic proteins, the β-hairpin conformation might affect several steps of the amyloid formation reaction (Fig. 1B): β-Hairpins may (i) constitute basic components of oligomers (on-pathway or off-pathway) and therefore trigger or suppress the nucleation of amyloid fibrils, (ii) bind to other types of oligomers and affect primary and/or secondary nucleation, (iii) bind to fibril ends and block fibril elongation, or (iv) bind to fibril surfaces and affect secondary nucleation. Many such aggregation-promoting or -inhibiting functions have been described for β-hairpins of several amyloidogenic proteins in the literature [8], [9], [14], [15], [17], [22], [24], [25], [26], [27], [28], [29], [30], [31], [32], [33], [34], [35], [36]. Yet there is no consensus on the impact of the β-hairpin conformation on amyloid formation, which warrants further investigations into the general and specific properties of β-hairpins.

**Fig. 2:**
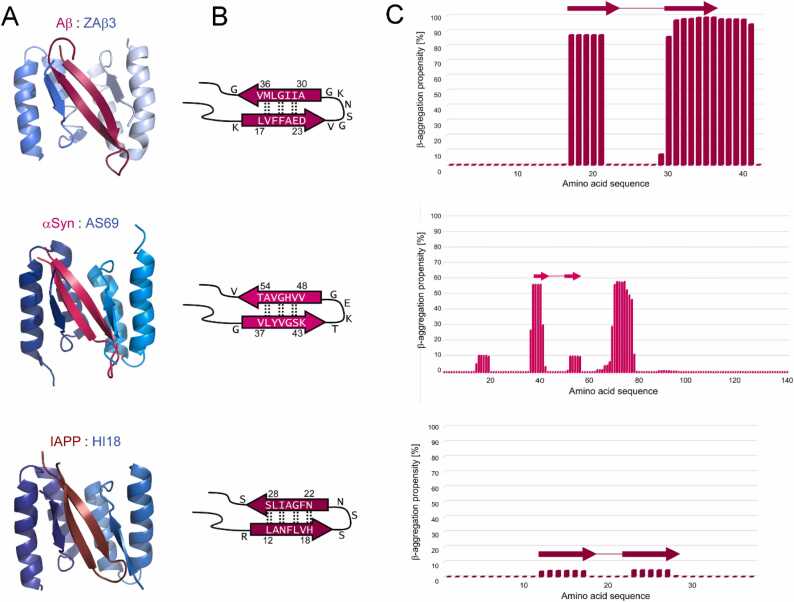
β-Hairpins of Aβ (top row), αSyn (middle row), and IAPP (bottom row). (A) NMR structures of local β-hairpin folds of Aβ (pdb 2OTK), αSyn (pdb 4BXL), and IAPP (pdb 5K5G), established upon coupled folding-binding to ZAβ3 or the β-wrapins AS69 and HI18, respectively [37], [38], [39]. (B) Corresponding β-hairpin registries. (C) β-Aggregation propensities of the amino acid sequences. The positions of the observed β-hairpins are indicated by arrows (β-strands) connected by a line (turn).

We have previously observed β-hairpins in amyloid-β (Aβ), α-synuclein (αSyn), and islet amyloid polypeptide (IAPP), amyloidogenic proteins associated with AD, PD, and T2D, respectively (Fig. 2) [37], [38], [39]. These β-hairpins are established upon binding to engineered binding proteins termed β-wrapins. β-Wrapins are selected from protein libraries based on ZAβ3, an affibody with high affinity for monomeric Aβ [37], [39], [40], [41], [42]. β-Hairpin structures of Aβ, αSyn and IAPP have been determined by NMR spectroscopy of their complexes with ZAβ3 and the β-wrapins AS69 and HI18, respectively (Fig. 2A) [37], [38], [39]. The β-hairpins are stabilized in the complexes by formation of an intermolecular β-sheet with two short β-stands contributed by the binding proteins, and by coverage of the their mostly hydrophobic side chains as the binding proteins wrap around the β-hairpins. Importantly, the β-hairpins of Aβ, αSyn and IAPP are established in sequence regions that according to MD simulations also populate β-hairpin conformation in the free state (Fig. 2B) [8], [11], [13], [23]. Interestingly, a single β-wrapin, denoted AS10, can bind all three amyloidogenic targets Aβ, αSyn and IAPP with sub-micromolar affinity, emphasizing the similarity of their β-hairpin regions [43].

Here, we aimed to identify further human proteins containing β-hairpin regions similar to Aβ, αSyn and IAPP. Such proteins might form oligomers or amyloid fibrils by themselves or they might affect oligomer or fibril formation of other proteins. β-Hairpin forming segments in the human proteome were predicted by sequence comparison with Aβ, αSyn and IAPP. Out of 2505 potential β-hairpin-forming amyloidogenic segments a test set of eight segments was characterized in peptide format, proving Thioflavin T (ThT)-positive aggregation for seven and β-hairpin formation for four of the candidates. Further analysis of one segment, which stems from prostatic acid phosphate (PAP), demonstrated a promoting effect on physiologically relevant PAP semen amyloid formation.

## 2 Materials and methods

### 2.1 Bioinformatics

The 64-bit Linux binary of the TANGO algorithm 2.3.1 [44], [45], [46] was downloaded from http://tango.crg.es. The annotated human proteome was acquired on the February 21st 2017 from Uniprot [47] using the query: *reviewed:yes AND organism:"Homo sapiens (Human) [9606]" AND proteome:up000005640* and downloading the amino acid sequences as a FASTA file (20,162 protein sequences). The sequences were then prepared for the TANGO algorithm by creating files with up to one thousand lines (TANGO limit) all having the following structure: "uniprot ID" N N 7 298 0.1 "aa_sequence", corresponding to running the TANGO algorithm on all sequences assuming no modifications of termini, T = 298 K, and an ionic strength of 0.1 M (default parameters of TANGO). These batch files were then supplied to a locally running instance of TANGO. For unknown reasons some sequences resulted in errors, both when run locally as well as when the amino acid sequences were supplied to the online version of TANGO, and they were therefore excluded from any analysis. The Uniprot IDs of the error causing sequences were: Q8NHP1, P22352, P59796, P18283, P07203, Q9C0D9, P36969, Q8IZQ5, Q9BVL4, Q9BQE4, Q8WWX9, P49908, P62341, P59797, Q9NZV5, P63302, Q9Y6D0, O60613, Q99611, Q9NNW7, Q86VQ6, Q8WZ42, P49895, Q92813, P55073, Q9NZV6, Q16881. A filter was applied based on the length of aggregation prone β-stretches, the length of the turn between the stretches, presence of aromatic residues, presence of hydrophobic residues, and the presence of glycine. The parameters were based on three previously identified aggregation-prone β-hairpins in Aβ, αSyn, and IAPP [37], [38], [39]. In particular, the following filter was developed and applied to the output from TANGO:

1. Only amino acids with a TANGO aggregation propensity > 1.06 were considered to be in β-stretch.
2. Only β-stretches between 5 and 23 amino acids long were considered for further analysis.
3. Only β-stretches connected to other β-stretches by 9 or fewer amino acids (i.e potential turns) were considered for further analysis.
4. Only β-stretches where the first (i.e. N-terminal strand) had the following properties were considered:

41. At least one aromatic residue.
42. A fractional content of the residues isoleucine, leucine, and valine between 0.32 and 0.5.

5. Only β-stretches where the second (i.e. C-terminal strand) had the following properties were considered:

51. Between 1 and 3 glycine residues.
52. A fractional content of the residues isoleucine, leucine, and valine between 0.32 and 0.61.

This resulted in 2505 potentially aggregation prone β-hairpins from 2098 protein sequences. The entire workflow is available as a Jupyter Notebook except the TANGO binary which should be obtained from the above mentioned source. Code and output sequences are available on zenodo under doi:10.5281/zenodo.8264535.

### 2.2 Peptides

Synthetic peptides were obtained either from peptides & elephants or from CASLO. To fully monomerize the peptides, 1 mg aliquots were solubilized in 1 mL hexafluoroisopropanol (HFIP), aliquoted in smaller amounts, lyophilized and stored at − 20 °C.

### 2.3 Cloning of the fusion constructs

Like other β-wrapins derived from the affibody ZAβ3, AS10 is a dimer of two identical subunits [43]. In the fusion constructs the two AS10 subunits and the β-hairpin-forming peptides were linked via flexible glycine-serine linkers in the following format: N-His_6_-AS10(subunit1)-(G_4_S)_3_-AS10(subunit2)-(G_4_S)_3_-HairpinPeptide-C. DNA fragments containing the sequence of the fusion constructs were obtained from Thermo Fisher Scientific and cloned into vector pET302/NT-His using In-Fusion HD EcoDry Cloning Kit (Takara Bio). The success of the cloning was verified by sequencing.

### 2.4 Expression and purification of the fusion constructs

Expression of the fusion constructs was done in *E. coli* JM109 DE3 cells. An overnight pre-culture was grown in 2YT medium supplemented with 100 µg/mL ampicillin at 37 °C and 180 rpm. For a subsequent overnight pre-culture, M9 minimal medium supplemented with 100 µg/mL ampicillin was inoculated with the 2YT pre-culture at an optical density of 0.05 and grown at the same conditions as the previous culture. An expression culture in M9 minimal medium supplemented with 100 µg/mL ampicillin, ^15^N-NH_4_Cl (1 g/L) and ^13^C_6_-glucose (2 g/L) was inoculated with the M9 pre-culture at an optical density of 0.05 and grown at 37 °C and 140 rpm until induction with Isopropyl-β-D-thiogalactopyranosid (IPTG), then incubated at 20 °C, 120 rpm for additional 20 h before harvesting. Cells were harvested by centrifugation at 5000 rpm for 10 min at 4 °C and frozen in loading buffer (50 mM Tris(hydroxymethyl)aminomethane (Tris), pH 8, 500 mM NaCl, 20 mM imidazole) with protease inhibitor (cOmplete EDTA-free Protease Inhibitor Cocktail, Roche). After thawing, cells were sonicated on ice twice for 5 min with the sonication probe MS-72, with an amplitude of 35% and a cycle of 3 s pulse and 5 s pause. Next, the cell debris was removed by centrifugation and the supernatant was applied to a NiNTA HisTrap FF column (GE Healthcare) in loading buffer. Protein was eluted with a mixture of loading buffer and elution buffer (50 mM Tris, pH 8, 500 mM NaCl, 500 mM imidazole) into a final concentration of 250 mM imidazole. The eluate was concentrated and applied to a size exclusion chromatography column (SEC Superdex 200 Increase 10/300 GL, GE Healthcare) in a sodium phosphate buffer (20 mM NaPi, pH 7.4, 50 mM NaCl). The purified fusion protein was frozen in liquid nitrogen and stored at − 80 °C.

### 2.5 Thioflavin T (ThT) aggregation assay

All ThT aggregation assays were done in Greiner 96-well half-area, clear bottom, low-binding plates. The measurements themselves were done in a BMG CLARIOstar plate reader at 37 °C and 300 rpm shaking in orbital mode with one glass bead (0.75–1 mm, Roth) per well. Wells with samples were always surrounded by wells filled with liquid, either other samples or 150 μl water, to counter potential artefacts due to evaporation. For characterization of the aggregation of the peptide test set, peptides were dissolved in 20 mM NaPi, pH 7.4, 50 mM NaCl, and added to the wells at final concentrations of 20 µM (CYP2W1, FibA, 3-PGDH, MIC26), 25 µM (TTC5, NDUFS7), 500 µM (NBPF1) or 550 µM (PAP) in triplicate samples of 80 μl. Final concentrations of 25 µM ThT and 0.04% sodium azide (NaN_3_) were added. The plate was sealed with sealing tape and put into the plate reader, heated to 37 °C, and ThT fluorescence was read in 1000 cycles of 300 s each using bottom optics with excitation at 448 nm (10 nm bandwidth) and emission at 482 nm (10 nm bandwidth). To investigate the effect of PAP(185−208) on the aggregation of PAP(248−286) and SEM1(86−107), the prepared peptides were dissolved in 50 mM Tris, pH 8.5. PAP(248−286) and SEM1(86−107) were kept at constant concentrations of 500 and 300 µM, respectively. The final concentration of PAP(185−208) was varied between 25 and 100 µM. Samples contained 40 µM ThT and 0.04% NaN_3_.

### 2.6 Atomic force microscopy (AFM)

For AFM imaging, 2 μl samples were taken out of the wells at the end of the ThT aggregation assays, put onto a freshly cleaved muscovite mica surface and dried during incubation for 10 min under the clean bench. Subsequently, the samples were washed with 100 μl ddH_2_O in three steps and dried with a stream of N_2_ gas. Imaging was performed in intermittent contact mode (AC mode) in a JPK NanoWizard 3 atomic force microscope using a silicon cantilever with silicon tip (OMCL-AC160TS-R3, Olympus) with a typical tip radius of 9 ± 2 nm, a force constant of 26 N/m and resonance frequency around 300 kHz. The images were processed using JPK DP Data Processing Software (version spm-5.0.84).

### 2.7 Isothermal titration calorimetry (ITC)

ITC was performed at 30 °C using a Microcal iTC200 calorimeter (GE Healthcare). AS10 was filled into the cell at a concentration of 30 µM in 20 mM sodium phosphate, pH 7.4, 50 mM NaCl. Peptides were titrated from the syringe in approximately 10-fold higher concentrations. Heats of post-saturation injections were averaged and subtracted from each injection to correct for heats of dilution and mixing. Dissociation constants were obtained from a nonlinear least-squares fit to a 1:1 binding model using MicroCal Origin.

### 2.8 NMR spectroscopy

The NMR spectra were acquired at 25 °C using VNMRS instruments (Varian) at proton frequencies of 800 and 900 MHz, each equipped with a cryogenically cooled Z-axis pulse-field-gradient (PFG) triple resonance probe. The sample buffer was 20 mM sodium phosphate, pH 7.4, 50 mM NaCl, supplemented with 10% D_2_O. For detection of complex formation, samples contained 100 μM ^15^N-labeled AS10, expressed and purified as described [39], [43], and a 20% excess of the unlabeled peptide. For determination of β-sheet registries, NMR samples contained ^13^C^15^N-labeled fusion constructs in concentrations between 360–700 μM. Backbone assignments were obtained using BEST-TROSY experiments [48]. Nuclear Overhauser effect (NOE) based distance restraints for β-sheet registry determination were derived from 3D (^1^H–^1^H-^15^N)-NOESY-HSQC (120 ms mixing time) and (^1^H–^13^Cali-^1^H)-HSQC-NOESY (100 ms mixing time) experiments. NMR data were processed using NMRPipe [49] and analyzed with CcpNMR [50].

### 2.9 Structure modeling

The structures of AS10 in complex with NDUFS7(104−129), PAP(185−208), or CYP2W1(176−204) were initially modeled using the structure of the AS10:Aβ complex [51] as a template. The residues of Aβ forming β-sheets with AS10 were first mutated to the residues of the investigated β-hairpin peptides forming β-sheets with AS10 according to the experimentally derived β-sheet registries. Subsequently, the loop residues of Aβ (VGSNKG) were deleted, and the loop residues of the peptides were modeled using Modeller [52]. The resulting structures were used as initial structures for 5-replicate MD simulations, which were performed using the same methods as in our previous studies on AS10 binding to Aβ, αSyn, and IAPP [51], [53]. Structural analysis comparing the simulated complex structures with experimental β-sheet registries and hydrogen atom distance constraints suggested that the MD simulations efficiently modeled the complex structures.

## 3 Results

### 3.1 Prediction of amyloidogenic β-hairpins

**Fig. 3:**
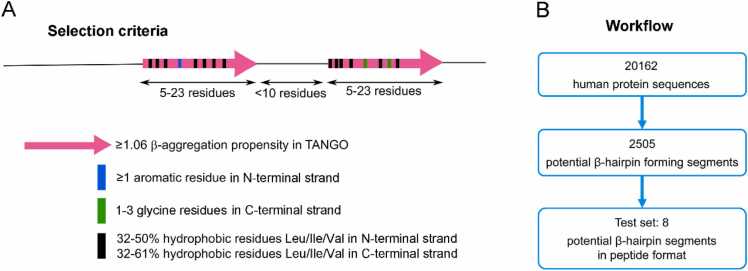
Criteria and workflow for prediction of amyloidogenic β-hairpins. (A) Protein segments were searched for that contained two segments of high β-aggregation propensity and an amino acid composition similar to the β-hairpins of Aβ, αSyn, and IAPP. (B) Numbers of protein sequences screened, predicted, and tested.

To identify potential β-hairpin-forming amyloidogenic segments in the human proteome, we aimed to search for protein segments with sequence properties similar to those of the β-hairpins of Aβ, αSyn and IAPP established in their complexes with the engineered binding proteins (Fig. 2). We therefore looked for commonalities among these three β-hairpin sequences that might serve as criteria in the search for further amyloidogenic β-hairpins. When comparing the sequences of the Aβ, αSyn and IAPP β-hairpins directly, no obvious sequence similarity exists (Fig. 2B). Moreover, secondary structure prediction did not reveal a common theme among the three sequences that could be used as a search pattern (Fig. S1). As we are specifically interested in β-hairpins capable of amyloid formation, amyloid propensity predictors are of interest. The TANGO algorithm predicts secondary structure and also calculates amyloid propensity, or β-aggregation propensity as TANGO denotes it, enabling identification of aggregation-prone regions (APRs) [44], [45], [46]. When applying TANGO to Aβ, αSyn and IAPP, a consistent pattern was observed where most of residues in the β-strands of the β-hairpins had high β-aggregation propensity scores and the parts corresponding to the turns had scores of zero (Fig. 2 C). Hence, a first search criterion was the presence of two regions with nonzero β-aggregation propensity scores separated by a segment of zero β-aggregation propensity. More specifically, sequences covering two stretches with between 5 and 23 consecutive residues with β-aggregation propensity scores ≥ 1.06 separated by maximally nine residues with a β-aggregation propensity score of zero were searched for (Fig. 3A). This resulted in 26,715 potential β-hairpins forming amyloidogenic regions distributed over 10,711 proteins out of a total of 20,162 analyzed human protein sequences. In other words, 53% of all sequences contained an average of 2.5 potential β-hairpin regions as judged by the TANGO search criterion. This high number is in line with the observation that the majority of human proteins contain multiple aggregation-prone regions (APRs) as assessed by computational predictions [54].

For a more stringent search pattern, additional requirements were imposed on the type of amino acids in the β-aggregation propensity-positive stretches, based on the specific sequence compositions of the Aβ, αSyn and IAPP β-hairpins. In particular, hydrophobicity and aromaticity were considered, as aromatic and hydrophobic amino acids have been implicated in amyloid formation [55], [56]. Slightly different requirements were placed on the N-terminal and C-terminal β-aggregation propensity-positive stretch to reflect the known properties of the Aβ, αSyn and IAPP β-hairpins. In the N-terminal stretch, at least one aromatic residue and a fractional content of the large aliphatic residues leucine, isoleucine, and valine between 0.32 and 0.5 of all residues were required. In the C-terminal stretch, between 1 and 3 glycine residues and a fractional content of the large aliphatic residues between 0.32 and 0.61 of all residues were required (Fig. 3A). The use of these additional requirements resulted in a reduction of hits to 2505 potential β-hairpin regions distributed over 2098 proteins (Fig. 3B). In other words, 10% of the human protein sequences contained an average of 1.2 potential β-hairpins. Code and output sequences of the prediction of amyloidogenic β-hairpins are available on zenodo under doi:10.5281/zenodo.8264535.

### 3.2 Characterization of a test set of potential amyloidogenic β-hairpin protein fragments

**Fig. 4:**
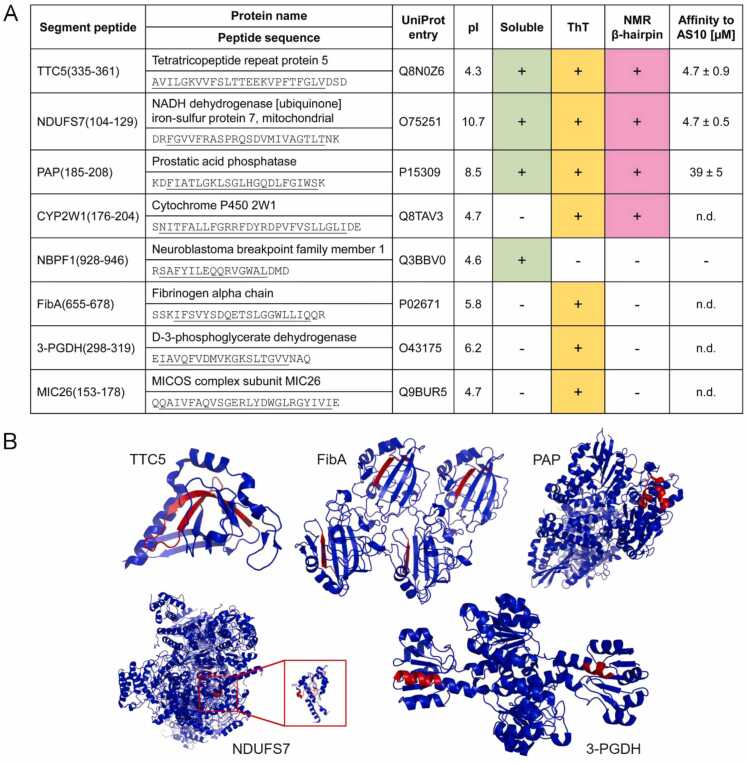
Test set of eight protein segments. (A) List of the investigated peptides. Underlined stretches in the peptide sequences correspond to the predicted amyloidogenic β-hairpins. A peptide was considered ‘soluble’ when no visible precipitation occurred upon dissolution of the lyophilized peptide in 20 mM NaPi, pH 7.4, 50 mM NaCl, to a final concentration of 20 µM. A peptide was considered ‘ThT’ positive, when increased ThT fluorescence was observed in aggregation assays performed in the peptide concentration range 20–550 µM for up to 85 h in 20 mM NaPi, pH 7.4, 50 mM NaCl. A peptide was considered to form an ‘NMR β-hairpin’ when the spectral fingerprint of β-hairpin formation was observed upon mixing 100 µM ^15^N-AS10 with a 20% molar excess of the respective peptide. Affinities were not determined (‘n.d.’) for peptides exhibiting precipitation. (B) Conformations of investigated segments (red) in the context of their source proteins. PDB codes 2XVS (TTC5), 1FZD (FibA), 1ND6 (PAP), 5XTB (NDUFS7), 6RJ3 (3-PGDH).

To evaluate whether the identified protein segments are indeed aggregation-prone and able to adopt β-hairpin conformation a test set of eight segments was investigated in peptide format (see Fig. 4A for source proteins, peptide names and sequences, and an overview of characterization results). We included in the test set the two segments that stem from proteins that are associated with the keyword ‘amyloid’ in Uniprot, namely fibrinogen alpha chain and prostatic acid phosphatase, as these might have an increased relevance in the context of amyloid formation. The other six segments were chosen randomly; however, transmembrane proteins were not considered. Apart from the predicted β-hairpin sequences, the peptides included up to three additional, mostly charged or polar amino acids at their termini, in accordance with the sequences of the source proteins, with the aim to a) increase peptide hydrophilicity and b) ensure that β-hairpin formation is not impeded by insufficient peptide length. In the sequence context of their source proteins, the segments adopt diverse secondary and tertiary structures (Fig. 4B). In one case, TTC5(335−361), the segment forms a β-hairpin in the source protein’s native fold. Here, we investigated the segments’ behavior when excised from the source protein.

**Fig. 5:**
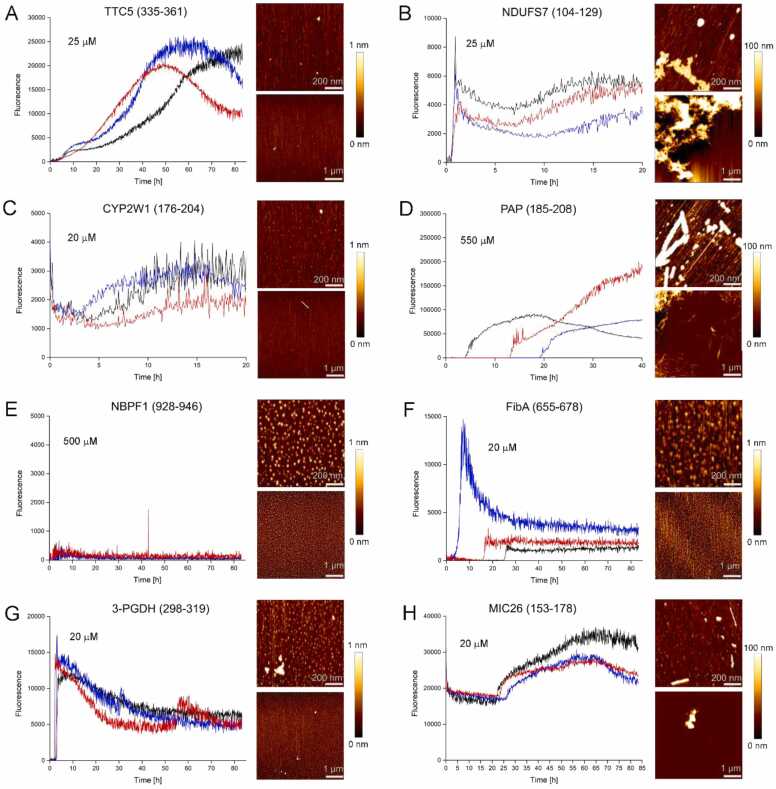
ThT aggregation assay and AFM. Left in panel, Time courses of ThT fluorescence upon incubation of the peptides at concentrations of 20 µM (CYP2W1, FibA, 3-PGDH, MIC26), 25 µM (TTC5, NDUFS7), 500 µM (NBPF1), or 550 µM (PAP), respectively, in 20 mM NaPi, pH 7.4, 50 mM NaCl. Three replicates per peptide are shown in black, blue, and red, respectively. Right in panel, AFM images of samples at the end of the aggregation assay.

When dissolved in aqueous buffer at µM concentrations at neutral pH, four of the eight peptides exhibited extensive precipitate formation indicative of low solubility (Fig. 4A). To test if any of the eight peptides form amyloid, the dye Thioflavin T (ThT), which shows dramatically increased fluorescence upon binding to amyloid fibrils [57], was added to 20–25 µM solutions of the peptides and time courses of ThT fluorescence were recorded (Fig. 5). Only in two cases, NBPF1(928−946) and PAP(185−208), no increase in fluorescence intensity was detected. When the peptide concentration was increased to 500–550 µM, PAP(185−208) exhibited ThT fluorescence (Fig. 5D), whereas NBPF1(928−946) remained ThT negative (Fig. 5E). For five peptides, ThT fluorescence was initially low but increased rapidly after a lag-time, which is characteristic of amyloid formation (Fig. 5A,B,D,F,G). Two of these five peptides, FibA(655−678) and 3-PGDH(298−319), belonged to the group showing immediate precipitate formation upon dissolution, indicating that they can form both ThT-positive as well as ThT-negative aggregates. For two further peptides exhibiting immediate precipitation, CYP2W1(176−204) and MIC26(153−178), ThT fluorescence was high from the beginning, indicating that ThT-positive structures had formed directly upon sample preparation (Fig. 5C,H). We aspired to image the aggregates at the end of the aggregation assays by AFM, but the amount of material on the substrate was typically sparse, which we attribute to a poor interaction of the comparatively hydrophobic peptides with the hydrophilic mica substrate. Nevertheless, fibrillar aggregates confirming amyloid formation were detected of NDUFS7(104−129), PAP(185−208), CYP2W1(176−204), and MIC26(153−178) (Fig. 5). Collectively, these initial experiments showed that only one of the eight peptides remained soluble, whereas the other seven formed aggregates with features typical for amyloids.

**Fig. 6:**
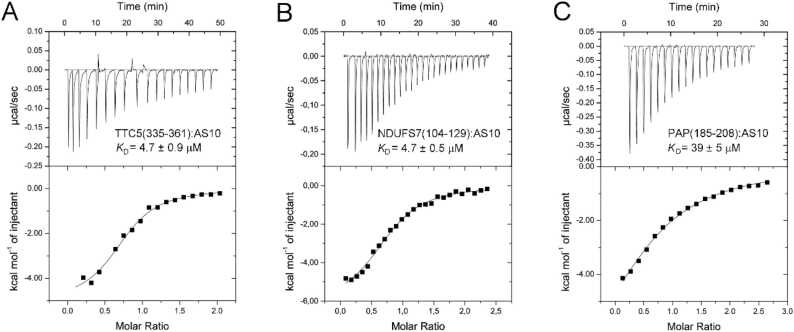
Binding of peptides to β-wrapin AS10 observed by ITC. Titrations of (A) 500 µM TTC5(335−361), (B) 300 µM NDUFS7(104−129), or (C) 300 µM PAP(185−208) into 30 µM AS10. The upper panels show the baseline-corrected instrumental response. The lower panels show the integrated data (filled squares) and the best fit of the parameters of a 1:1 binding model (continuous line).

We next applied ITC to test if the peptides have affinity for β-wrapin AS10, which binds Aβ, αSyn and IAPP each with sub-micromolar affinity and concomitantly induces β-hairpin conformation [43]. This was investigated only for the four peptides that did not precipitate in aqueous buffer at neutral pH, as heats resulting from precipitate formation or dissolution would superpose the ITC signal that stems from binding. For the three peptides that exhibited an increase in ThT fluorescence in the course of the aggregation assays, TTC5(335−361), NDUFS7(104−129), and PAP(185−208), heat signals in agreement with 1:1 binding were observed, yielding dissociation constants of 4.7 ± 0.9 µM, 4.7 ± 0.5 µM, and 39 ± 5 µM, respectively (Figs. 4A and 6). In contrast, heat signals were not observed for the peptide NBPF1(928−946) that was soluble and did not show ThT fluorescence.

**Fig. 7:**
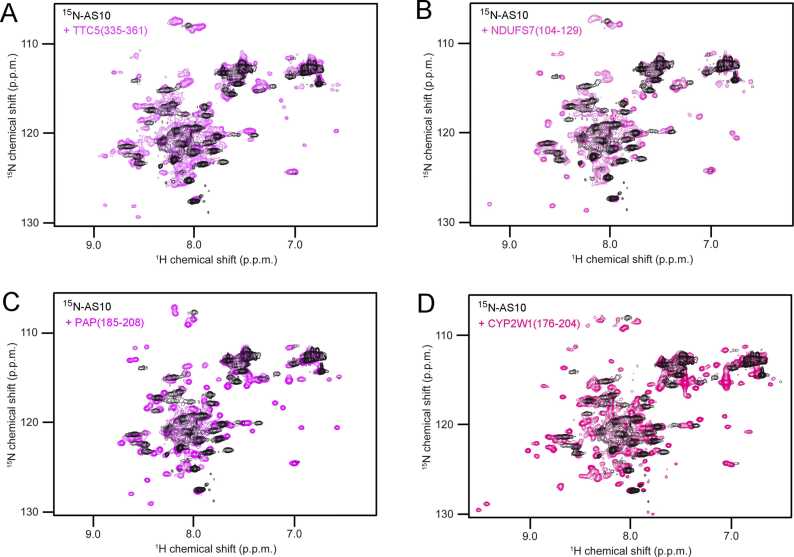
NMR spectroscopy indicates β-hairpin formation upon AS10 binding. ^1^H–^15^N HSQC NMR spectra of ^15^N-AS10 in the absence (black) or presence (magenta to pink red color range) of a slight excess of unlabeled (A) TTC5(335−361), (B) NDUFS7(104−129), (C) PAP(185−208), or (D) CYP2W1(176−204), respectively.

To test for β-hairpin formation in the peptides upon binding to AS10 we performed NMR spectroscopy. ^1^H–^15^N HSQC NMR spectroscopy using ^15^N-labeled AS10 (^15^N-AS10) and unlabeled binding partner leads to characteristic changes upon β-hairpin folding coupled to binding. These include a great increase in resonance dispersion, appearance of four amide proton resonances in the glycine region (^15^N chemical shift <110 ppm) stemming from Gly-13 and Gly-14 in the two AS10-subunits, as well as appearance of amide proton resonances with shift values typical for β-sheet conformation (high ^1^H and high ^15^N chemical shifts) [43]. This signature was observed here when 100 µM ^15^N-AS10 was mixed with a 20% molar excess of one of the three peptides TTC5(335−361), NDUFS7(104−129) or PAP(185−208), which possess affinity for AS10 according to ITC (Fig. 7A-C). Interestingly, this signature was also observed for CYP2W1(176−204), which formed precipitates upon dissolution, indicating that this peptide can also partition into a 1:1 complex with AS10 (Fig. 7D). In contrast, no change in the ^1^H–^15^N HSQC NMR spectrum of ^15^N-AS10 was observed upon addition of the other four peptides, which is in line with the absence of AS10 affinity for NBPF1(928−946) demonstrated by ITC and with the insolubility of FibA(655−678), 3-PGDH(298−319), and MIC26(153−178). It is possible the latter three peptides would engage in an interaction with AS10 and adopt β-hairpin structure when brought in contact with AS10 before precipitating into aggregates; however, this was not investigated further.

**Fig. 8:**
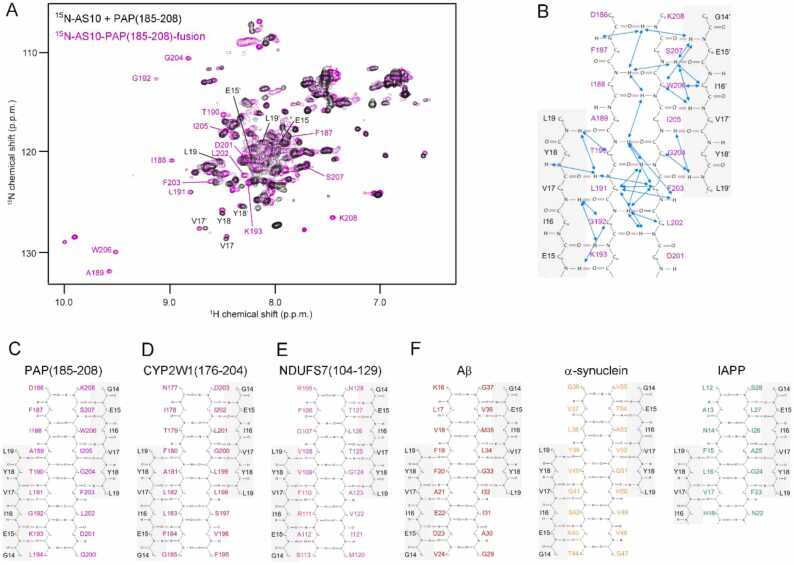
**Determination of β-sheet registries.** (A) Overlay of the ^1^H–^15^N HSQC NMR spectra of ^15^N-AS10 in the presence of unlabeled PAP(185−208) (black) and of the ^13^C,^15^N-labeled AS10-PAP(185−208) fusion construct (magenta). Assignments of backbone amide resonances stemming from the β-sheet were obtained by NMR spectroscopy of the fusion construct and are displayed in black for AS10 residues and in magenta for PAP(185−208) residues. (B) NOE contacts (blue arrows) involving backbone NH and Hα protons allow identification of the β-sheet registry in the PAP(185−208):AS10 complex. Two AS10 β-strands (gray background, black residue labels) flank the β-hairpin formed by PAP(185−208) (white background, magenta residue labels). (C)-(F) Comparison of β-hairpin registries determined in this work (C-E) with those identified previously in Aβ, αSyn, and IAPP (F) [37], [38], [39].

To determine the registries of the β-hairpins of TTC5(335−361), NDUFS7(104−129), PAP(185−208), and CYP2W1(176−204) formed in complex with AS10, we performed NMR spectroscopy on the ^13^C,^15^N-labeled complexes of peptides with AS10. To circumvent cost-intensive chemical synthesis of isotopically labeled peptides, fusion constructs of AS10 at the N-terminus and the respective peptide at the C-terminus, linked via a flexible (G_4_S)_3_ linker, were produced recombinantly. For NDUFS7(104−129), PAP(185−208), and CYP2W1(176−204), the backbone amide resonances of ^15^N-labeled AS10 bound to the unlabeled peptides (Fig. 7B-D) were recovered in the ^13^C,^15^N-labeled fusion constructs, demonstrating that the structure of the complex is not affected by the fusion (Fig. 8A). In contrast, this was not true for the AS10 fusion construct of TTC5(335−361), which prohibited determination of the β-hairpin registry in this case. For registry determination, the resonances stemming from the β-hairpin motif were assigned by standard triple resonance heteronuclear NMR techniques and characteristic NOE contacts involving NH and Hα protons in the backbones of the peptides and of AS10 were identified. For all three peptides, the NOE contacts were compatible with only one unique β-hairpin registry (Fig. 8B and S2). The β-hairpin registries determined for PAP(185−208), CYP2W1(176−204), and NDUFS7(104−129) are shown in Fig. 8C-E along with those of Aβ, αSyn and IAPP (Fig. 8F).

**Fig. 9:**
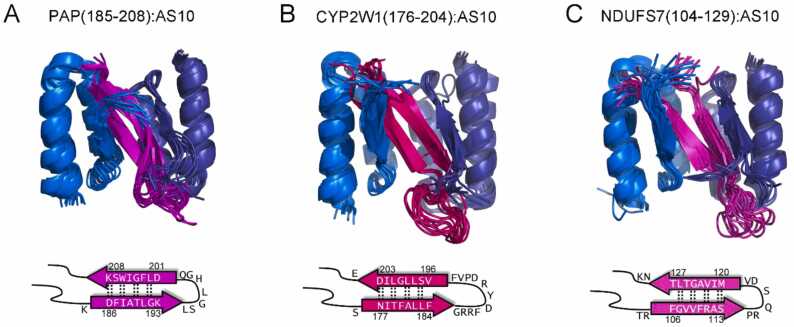
Computationally predicted structural models of β-hairpins in complex with AS10. Models were initially built based on the common β-sheet architecture of the newly identified β-hairpin complexes and the known β-hairpin complexes of Aβ, αSyn and IAPP. The 10 structures of lowest energy in subsequent MD simulations are displayed. The corresponding coordinate files are available in the supplementary material.

As the general architecture of all six intermolecular β-sheets was identical in all the cases (Fig. 8C-F), we modeled the structures of the AS10 complexes of PAP(185−208), CYP2W1(176−204), and NDUFS7(104−129) using the known structures of β-wrapins with Aβ, αSyn and IAPP as starting point. Specifically, the template used for initial modeling of the AS10 complexes of the peptides was the structure of the Aβ:AS10 complex [51]. We first mutated the Aβ residues that form the two β-strands to the residues of the peptides at the corresponding position in the experimentally derived β-sheet registries. Subsequently, the loop residues of Aβ (VGSNKG) were replaced by the loop residues of the individual peptides, modeled using Modeller [52]. The resulting structures were used as initial structures for MD simulations performed analogous to our previous studies [51], [53], yielding snapshots of the AS10 complexes of PAP(185−208), CYP2W1(176−204), and NDUFS7(104−129) which were generally in agreement with the experimental constraints (Fig. 9). Files containing coordinates of the ten snapshots of lowest energy for each complex are available in the supplementary material of this article.

Taken together, the characterization of the test set of eight protein segments identified in the prediction showed that the majority of them formed ThT-positive structures. For four of the eight segments, β-hairpin formation in complex with AS10 analogous to that observed for Aβ, αSyn and IAPP was confirmed.

### 3.3 PAP(185-208) triggers semen amyloid formation

The herein identified protein segments with capacity to adopt β-hairpin conformation are usually incorporated into the native fold of their source protein, which prohibits amyloid formation (Fig. 4B). However, when liberated from the native fold, they might be able to form amyloid and gain new biological activities. For example, prostatic acid phosphatase (PAP) is synthesized in the prostatic gland and secreted into the semen, where it is proteolytically cleaved into many fragments [58]. Two of these fragments, PAP(248−286) and PAP(85−120) are known to exist as amyloids in the seminal plasma [59], [60]. Similarly, semenogelin-1 (SEM1) and semenogelin-2 (SEM2) are cleaved into fragments that also form semen amyloids [61], [62]. The semen amyloids belong to the class of functional amyloid, as they fulfill beneficial roles in the selection of healthy sperms [63].

**Fig. 10:**
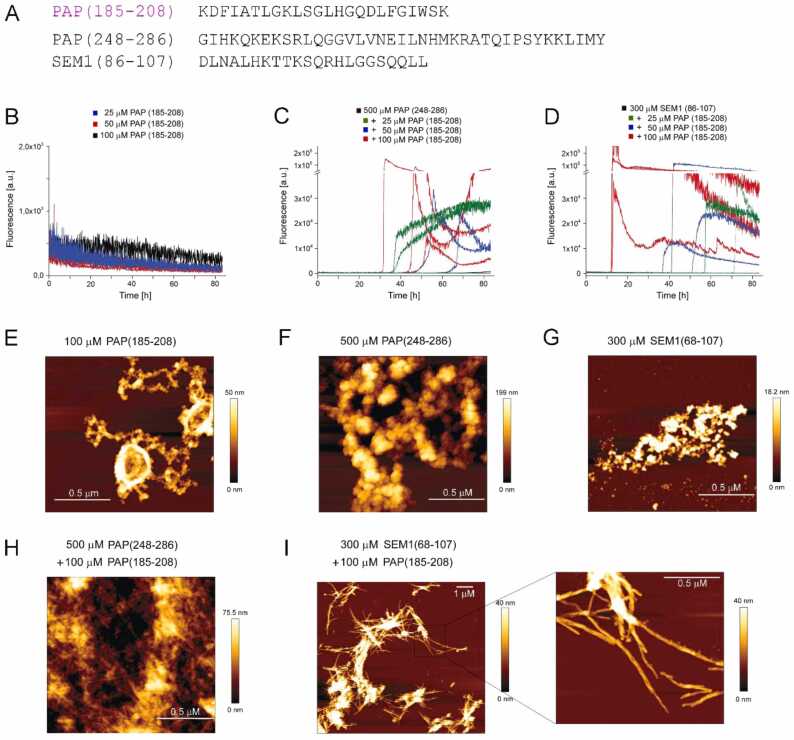
PAP(185−208) triggers semen amyloid formation. (A) Amino acid sequences of the identified β-hairpin-forming amyloidogenic segment PAP(185−208) and the semen amyloid fragments PAP(248−286) and SEM1(86−107). (B)-(D) Time courses of ThT fluorescence of (B) PAP(185−208) alone, (C) PAP(248−286) in absence and presence of PAP(185−208), and (D) SEM1(86−107) in absence and presence of PAP(185−208). (E)-(I) AFM images at the end of the aggregation assays of the individual peptides (E-G) and of the co-incubations of PAP(185−208) with (H) PAP(248−286) or (I) SEM1(86−107).

Here we tested if the β-hairpin-forming PAP segment PAP(185−208) identified above can modulate amyloid formation of PAP(248−286) or SEM1(86−107), as these interactions might occur in the seminal plasma (Fig. 10). PAP(185−208) at concentrations up to 100 µM did not exhibit ThT fluorescence in the time frame of the aggregation assay (Fig. 10B); concentrations > 0.5 mM, however, were sufficient for amyloid formation (Fig. 5D). PAP(248−286) and SEM1(86−107) did not exhibit ThT fluorescence in the time frame of the aggregation assays at concentrations of 500 µM and 300 µM, respectively (Fig. 10C,D), in line with the high concentrations (>1 mM) applied in previous studies of in vitro amyloid formation of these fragments [60], [61]. Nevertheless, even in the absence of ThT fluorescence at moderate peptide concentrations, aggregates of amorphous shape were detected by AFM of PAP(185−208), PAP(248−286), and SEM1(86−107) (Fig. 10E-G). When low concentrations of PAP(185−208) were added to aggregation assays of 500 µM PAP(248−286) or 300 µM SEM1(86−107), ThT time traces featuring a lag-time and a phase of rapid growth indicative of amyloid formation were obtained (Fig. 10C,D). The corresponding AFM demonstrated a shift to fibrillar aggregate morphology (Fig. 10H,I). The data indicates that PAP(185−208) can trigger the formation of semen amyloids, at concentrations where PAP(185−208) alone does not form amyloid.

**Fig. 11:**
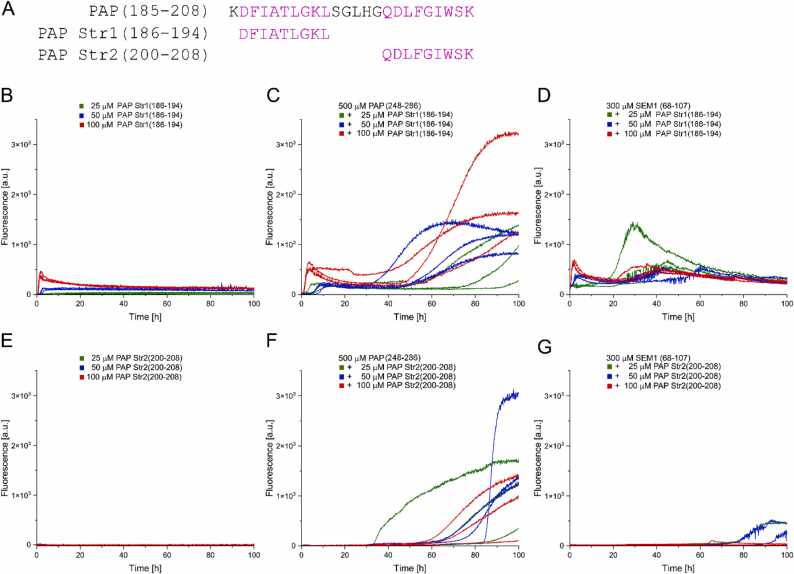
Individual β-strand segments of PAP(185−208) trigger semen amyloid formation. (A) Amino acid sequences of the β-hairpin-forming amyloidogenic segment PAP(185−208) and the two constituent β-strand segments PAP Str1(186−194) and PAP Str2(200−208). (B)-(D) Time courses of ThT fluorescence of (B) PAP Str1(186−194) alone, (C) PAP(248−286) in presence of PAP Str1(186−194), and (D) SEM1(86−107) in presence of PAP Str1(186−194). (E)-(G) Time courses of ThT fluorescence of (E) PAP Str2(200−208) alone, (F) PAP(248−286) in presence of PAP Str2(200−208), and (G) SEM1(86−107) in presence of PAP Str2(200−208).

We next tested whether promotion of semen amyloid formation is an activity exhibited by the individual β-strands constituting the β-hairpin forming segment, or if it requires the entire β-hairpin segment PAP(185−208). Therefore, the two β-strand peptides PAP Str1(186−194) and PAP Str2(200−208) were investigated (Fig. 11A). PAP Str1(186−194) in the concentration range of 25–100 µM showed a concentration-dependent increase in ThT fluorescence within minutes to a few hours, indicating that it forms amyloid on its own (Fig. 11B). This is in contrast to PAP Str2(200−208) (Fig. 11E) and to the complete β-hairpin segment PAP(185−208) (Fig. 10B), which do not exhibit ThT fluorescene in this concentration range. When PAP Str1(186−194) was added to aggregation assays of 500 µM PAP(248−286) or 300 µM SEM1(86−107), ThT time traces exhibited additional phases of rapid growth (Fig. 11C,D), similar to those observed when the entire β-hairpin segment PAP(185−208) was added to 500 µM PAP(248−286) or 300 µM SEM1(86−107) (Fig. 10C,D). Similarly, addition of PAP Str2(200−208) to 500 µM PAP(248−286) or 300 µM SEM1(86−107) induced increases in ThT fluorescence, albeit with somewhat longer lag-times (Fig. 11F,G). This suggests that promotion of semen amyloid formation does not require the entire β-hairpin segment PAP(185−208), but that both constituent β-strands are sufficient to trigger amyloid formation. We conclude that cross-interaction of the herein identified β-hairpin forming segment PAP(185−208) with PAP(248−286) and SEM1(86−107) can promote semen amyloid formation, which is an activity exerted by both of its constituent β-strand segments.

## 4 Discussion

In this study we aimed to identify protein segments with similarity to Aβ, αSyn and IAPP, three highly amyloidogenic proteins with important roles in protein misfolding diseases. Starting point was the common formation of β-hairpins in all three proteins in regions that are critical for amyloid formation [37], [38], [39]. These β-hairpins are stabilized upon binding to engineered binding proteins, have a common architecture, and one binding protein, β-wrapin AS10, binds all three proteins with sub-micromolar affinity [43]. The search for proteins with propensity to form similar β-hairpins might identify further molecules that contribute to disease processes and may improve our understanding of the roles of β-hairpins in amyloid formation. By combining the capacity of the TANGO algorithm to predict APRs with the general architecture of β-hairpins and some specific features of the amino acid composition of the Aβ, αSyn and IAPP β-hairpins, we filtered the human proteome and obtained 2505 potential β-hairpin regions distributed over 2098 proteins. The characterization of the test set of eight protein segments showed that this approach could indeed identify protein segments that form amyloid and can adopt β-hairpin conformation. The test set included one peptide, NBPF1(928−946), that did not form amyloid and remained soluble, showing that the filtered sequences have to be analyzed case-by-case. The test data set was not large enough to derive further factors deciding if or if not a sequence will form ThT-positive aggregates and bind to AS10 in a β-hairpin conformation. However, the wide range of pI values of the identified peptides indicates that no specific charge state is required (Fig. 4A). The peptides within the test set with highest affinity for AS10 achieved a *K*_D_ of 5 µM, which is an order of magnitude higher than the *K*_D_s of Aβ, αSyn and IAPP [43]. The higher affinity of Aβ and αSyn is not surprising, as the generation of AS10 included steps for selection for Aβ binding and affinity maturation for αSyn. The high affinity of IAPP, on the other hand, is remarkable, as it was not involved in the selection [43].

The search strategy for amyloidogenic β-hairpins was governed by sequence similarity with the β-hairpin segments of Aβ, αSyn and IAPP. Altered search criteria, such as higher or lower TANGO aggregation propensities, different lengths of β-strand or turn regions, or different amino acid compositions, will result in variable lists of protein segments, which might be as relevant or even more relevant than the list generated here. As a matter of principle, a potential relevance of the identified β-hairpin segments has to be evaluated case-by-case. The segments are usually incorporated into the source protein structure, lacking that conformational flexibility that is required for folding into a β-hairpin or into an amyloid structure (Fig. 1). However, the segments may gain this flexibility during their lifetimes, as described above for PAP, which is cleaved into fragments by proteases in the seminal fluid. PAP fragments that include the segment PAP(185−208) are likely present in the seminal fluid, where they may trigger the formation of semen amyloids built from PAP(248−286) and SEM1(86−107), as suggested by the aggregation assay data (Fig. 10). Investigation of the two APR peptides that together constitute the PAP(185−208) β-hairpin segment revealed that the individual APRs were both able to trigger semen amyloid formation, i.e., PAP(185−208) combines two β-strand peptides that can both trigger cross-amyloid formation on their own (Fig. 11). Further case-by-case experiments are required to elucidate which potential novel properties the β-hairpin segments gain by combining two APRs. Interestingly, the ability of PAP Str1(186−194) to form ThT-positive aggregates on its own (Fig. 11B) does not seem to be required for triggering heterologous amyloid formation, as the complete β-hairpin peptide PAP(185−208) does not form ThT-positive aggregates alone but still promotes semen amyloid formation.

In conclusion, this study has identified a large number of potential β-hairpin-forming amyloidogenic protein segments in the human proteome which may have important roles in amyloid formation and which may serve as a resource to study general and specific effects of β-hairpin conformers on amyloid formation.

## CRediT authorship contribution statement

Conceptualization, LFH, EDA, WH; Investigation, Methodology, LFH, EDA, AAO, TK, WS, MMW, MSc, PT, MSt; Software, EDA; Funding acquisition, Supervision, PT, WH; Project administration, WH; Writing – original draft, LFH, EDA, AAO, PT, WH; Writing – review & editing, LFH, EDA, AAO, TK, WS, MMW, MSc, DW, PT, MSt, WH.

## Declaration of Competing Interest

The authors declare no conflicts of interest.
