# Supplementary material for: Sequence-based identification of amyloidogenic β-hairpins reveals a prostatic acid phosphatase fragment promoting semen amyloid formation
Source: Comput Struct Biotechnol J. 2023 Dec 21;23:417–30. doi: 10.1016/j.csbj.2023.12.023 (PMC10787225; doi:10.1016/j.csbj.2023.12.023)
Supplement: Supplementary file 1 — Supplementary material [file mmc1.docx]

**Supporting Information**


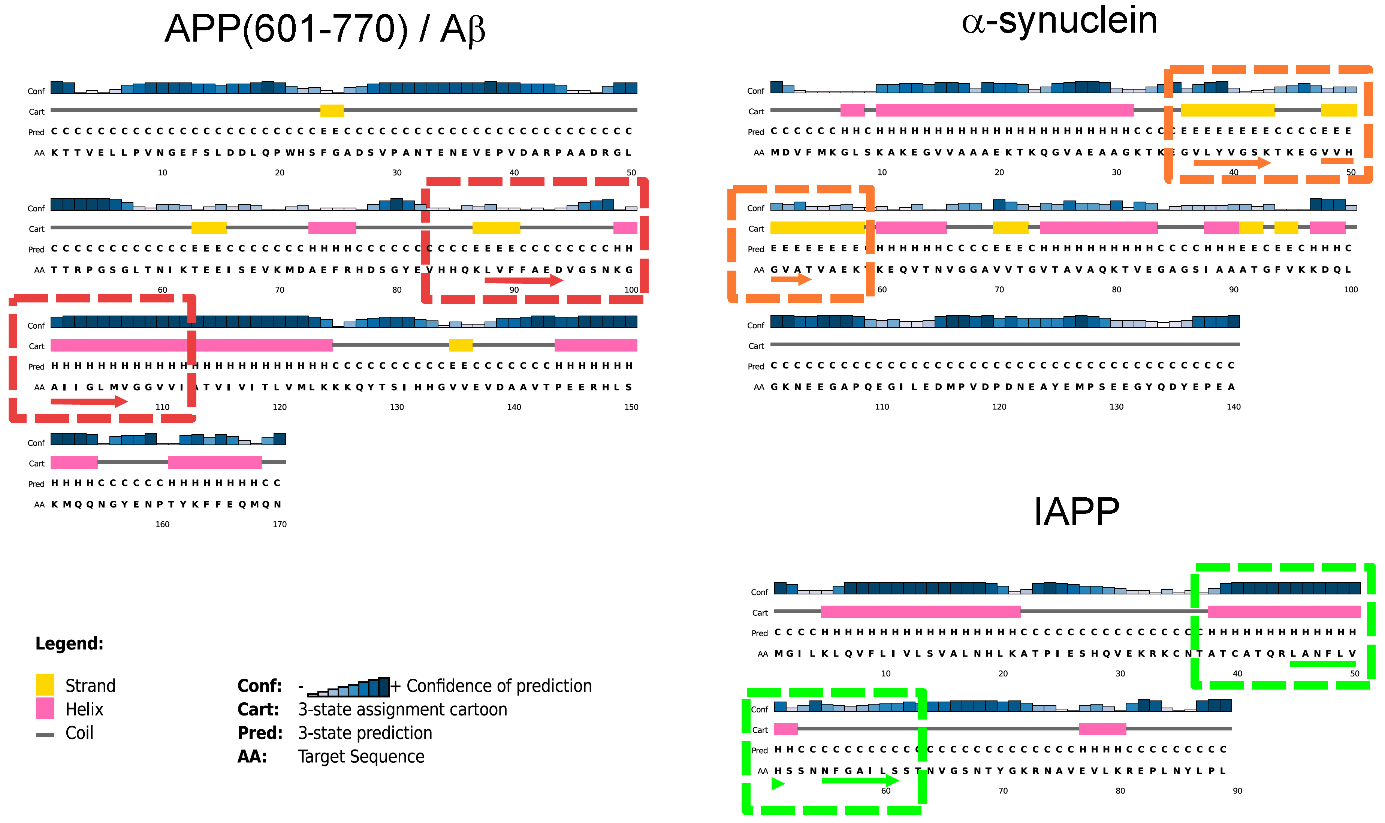


**Fig. S1. Secondary structure prediction does not suggest commonalities between the β-hairpin regions of Aβ, αSyn and IAPP.** Secondary structure prediction was performed with the algorithm PSIPRED, for Aβ and IAPP in the sequence context of their precursor proteins. The β-hairpin-forming regions are highlighted in dashed boxes and β-strand positions are indicated by arrows. For αSyn, β-strand propensity was lining up well with the β-strands in its β-hairpin. In contrast, this was not observed for Aβ and IAPP.


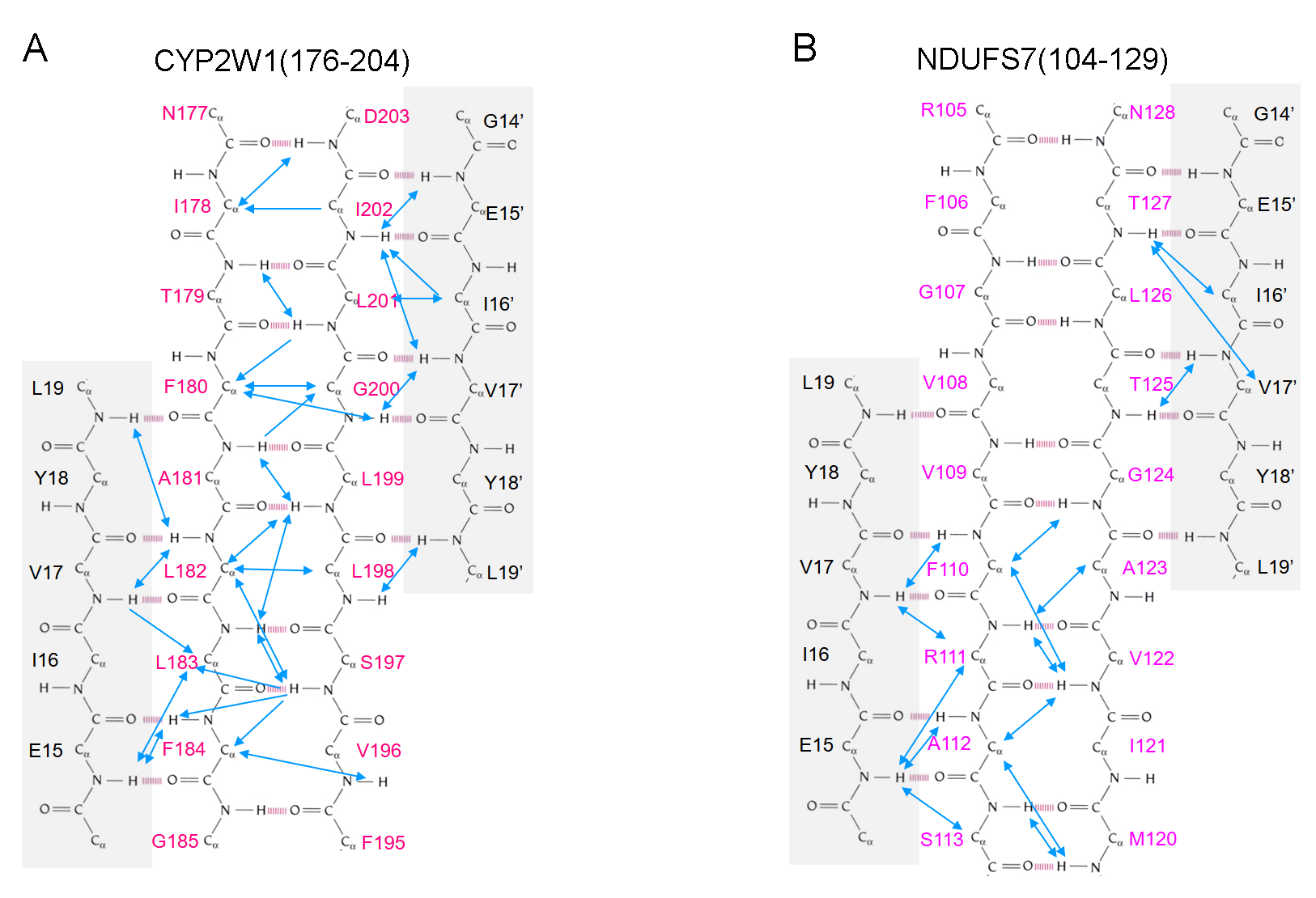


**Fig. S2. β-Sheet registries of CYP2W1(176-204) and NDUFS7(104-129).** β-Sheet registries were determined from NOE contacts involving backbone NH and Hα protons (blue arrows). Two AS10 β-strands (gray background, black residue labels) flank the β-hairpins formed by (A) CYP2W1(176-204) (white background, pink red residue labels) or (B) NDUFS7(104-129) (white background, magenta residue labels).
